# The Correlation of Burnout and Optimism among Medical Residents

**DOI:** 10.7759/cureus.6860

**Published:** 2020-02-03

**Authors:** James B Fowler, Brian Fiani, John W Kiessling, Yasir R Khan, Chao Li, Syed A Quadri, Deependra Mahato, Javed Siddiqi

**Affiliations:** 1 Neurosurgery, Desert Regional Medical Center, Palm Springs, USA; 2 Neurosurgery, Massachusetts General Hospital Harvard Medical School, Boston, USA

**Keywords:** medical education, burnout, resident burnout, optimism, residency

## Abstract

Introduction

Burnout is common among clinical specialties and has implications on the residents’ well-being and mental health. Evidence suggests that optimism and burnout are correlated, but research has not focused on the applicability to medical residents. The objective of the study was to define burnout in residents and correlate it with optimism.

Methods

The authors conducted a correlational, prospective cross-sectional study using self-reported single item burnout (1-10) and Life Oriented Test-Revised (LOT-R) (0-24) survey instruments among residents of neurosurgery, neurology, internal medicine, family medicine and emergency medicine at a community-based hospital. Residents were asked to fill out the survey once in the 2018 academic year and once again in the 2019 academic year. Burnout and optimism scores were correlated and compared across subgroups for each year.

Results

There was no statistical significance found amongst any subgroups in burnout in 2018 but significance was found for both internal medicine (t = 3.74, p = 0.001) and neurosurgery (t = -3.07, p = 0.01) in 2019. Mean burnout increased from 2018 to 2019 from 4.39 to 5.1. Optimism remained constant from 2018 to 2019 (16.7 and 16.76, respectively) and there was no difference across subgroups. There was a statistically significant negative correlation between burnout and optimism in both 2018 (r = -0.364, p = 0.006) and 2019 (r = -0.39, p = .001).

Conclusion

Burnout and optimism are negatively correlated. Although burnout increased over time, optimism remained constant indicating the stability of this trait and implication for future interventions.

## Introduction

Burnout has been at the forefront of public attention and medical education research over the previous decades. Burnout is a psychological term that refers to long-term exhaustion and diminished interest in work with symptoms similar to those of clinical depression. The term “burnout” was coined in the 1970s by the American psychologist Herbert Freudenberger who used it to describe the consequences of severe stress and high ideals experienced by people working in “helping” professions [1]. It has been defined further as a work-related syndrome to include emotional exhaustions, depersonalization and reduced personal accomplishment. Emotional exhaustion consists of feeling overwhelmed by job demands and depletion of emotional resources, depersonalization involves feelings of cynicism towards patients and reduced personal accomplishment is the decline in feelings of work competence and achievement [2].

Burnout involves both circumstantial and existential components. Circumstantial burnout stems from self-limited circumstances and environmental triggers, while existential burnout stems from a loss of meaning in medicine and an uncertain professional role [3].

Numerous risk factors and protective factors have helped predict the effect of burnout on medical residents which have helped to develop interventions aimed at decreasing burnout. However, despite the substantial research on burnout it continues to remain high among medical residents with negative effects on job performance and resident well-being, necessitating further research to develop interventions.

Optimism is defined as a disposition or tendency to look on the more favorable side of events or conditions and to expect the most favorable outcome [4]. Although there is evidence to suggest that optimism and burnout are correlated, there has been no research that has focused its applicability to medical residents [5]. The focus of the study is to define burnout in residents and correlate it with optimism.

## Materials and methods

A correlational, prospective cross-sectional study using self-reported single item burnout and Life Oriented Test-Revised (LOT-R) survey instruments were distributed to medical residents at Desert Regional Medical Center, a community hospital in Palm Springs, CA, to assess the burnout and optimism of residents. The study was reviewed and approved by the Institutional Review Board prior to distribution of surveys.

Participants and procedures

Participants included all residency programs at the hospital including neurosurgery, neurology, internal medicine, family medicine and emergency. The surveys were given to the respective department’s residency coordinator who distributed the survey to the residents during conferences or lectures. The purpose of the study was explained to the residents and they were asked to voluntarily participate in the study. Aggregate reporting of the data assured to enhance confidentiality and accurate reporting by the respondents. The completed forms were inputted from paper data by the research coordinator who calculated the optimism score from the LOT-R test. Residents were asked to fill out a survey once in 2017-2018 calendar year and then again in 2018-2019 calendar year.

Surveys

The distributed surveys consisted of three pages. The first page was an information sheet to inform the participant of the voluntary nature of the study. The second page included self-reported demographics including gender (male; female; prefer not to disclose), age (22-27; 28-32; 33-37; 38-43; 44-49; 49+), specialty (neurosurgery; neurology; family medicine; internal medicine; emergency medicine) and year of training (PGY-1; PGY-2; PGY-3; PGY-4; PGY-5; PGY-6; PGY-7). There was also a 10-point scale for participants to rate their current level of “burnout” as defined by the participants understanding of the term. A score of one reflected no burnout and a score of 10 reflected extreme burnout [6, 7].

The final page consisted of the Life Oriented Test-Revised (LOT-R). The LOT-R consisted of 10 questions. It is a measure of optimism versus pessimism [8]. Of the 10 items, three items measure optimism, three items measure pessimism, and four items serve as fillers. Respondents rate each item on a four-point scale: 0 = strongly disagree, 1 = disagree, 2 = neutral, 3 = agree, and 4 = strongly agree. Items three, seven, and nine were reverse scored. Items two, five, six, and eight were fillers and were not scored. Score of 24 reflected highly optimistic and zero no optimism.

Data analysis and statistics

Data analysis and optimism score derived from the LOT-R was completed by the research coordinator. Forms missing any value were not included in the data analysis. A burnout score and optimism score were calculated for each individual participant. Descriptive statistics were computed for demographics, burnout score and optimism score during each academic year separately to include mean and standard deviation. Response rate was calculated for a percentage of completed survey over the total number of residents in the hospital during that academic year. The means, standard deviation and one-way sample t-test for burnout were calculated for each subgroup in 2018 and 2019. The means, standard deviation and one-way sample t-test for optimism were also calculated for each subgroup in 2018 and 2019. Correlational analysis was completed to examine the relationship between burnout and optimism using Pearson’s correlation coefficient.

Subgroup analysis included gender, age, specialty and post-graduate year. Paired t-tests were performed to analyze the difference of the means between the demographic variables, in the context of burnout and optimism, separately for 2018 and 2019.

Data was pooled for 2018 and 2019 to compare differences between the subgroups PGY and specialty. The change in burnout score and optimism score from 2018 to 2019 was calculated by taking the difference. Analysis of variance was used to analyze the difference of means of the pooled data. Burnout score and optimism score were each analyzed separately.

All analyses were performed using SPSS Version 17 (IBM Corp., Released 2011, Armonk, NY). For all tests, alpha (α) was set at 0.05 with a confidence interval at 95%.

## Results

Population

The survey was completed by 58 out of 86 (67.44%) medical residents in 2018 and 75 out of 110 (68.18%) medical residents in 2019. The 2019 respondents included 2018 respondents also; though it was an anonymous survey, it is not certain how many 2018 respondents also filled the forms in 2019. Respondents were enrolled in neurosurgery, neurology, internal medicine, family medicine and emergency medicine in both 2018 and 2019. Two surveys from 2018 and four surveys from 2019 were not included in the statistical analysis as those forms had at least one variable missing or blank which brought the completed forms to 56 and 71 in 2018 and 2019, respectively.

In 2018, there were 33 males, 21 females and two preferred not to disclose their gender. Eight were within the age bracket of 22-27, 31 within the age bracket of 28-32, 12 within the age bracket of 33-37 and five were within the age bracket of 38-43. Eleven respondents were enrolled in emergency medicine, 13 in family medicine, 16 in internal medicine, nine in neurology and seven in neurosurgery. Twenty-three were in their first year of training (PGY-1), 25 were enrolled in PGY-2, seven were PGY-3 and one was PGY-4 (Table 1).

**Table 1:** Demographics for 2018 and 2019. Five residency programs were represented across five post-graduate training years over the duration of the study. PGY: Postgraduate Year

Category	Subgroup	2018	2019
Gender			
	Male	33	39
	Female	21	31
	Prefer not to disclose	2	1
Age			
	22-27	8	4
	28-32	31	44
	33-37	12	18
	38-43	5	3
	44-49	0	2
	49+	0	0
Specialty			
	Emergency Medicine	11	20
	Family Medicine	13	12
	Internal Medicine	16	19
	Neurology	9	10
	Neurosurgery	7	10
PGY			
	PGY-1	23	21
	PGY-2	25	23
	PGY-3	7	19
	PGY-4	1	7
	PGY-5	0	1
	PGY-6	0	0
	PGY-7	0	0
TOTAL		56	71

In 2019, there were 39 males, 31 females and one preferred not to disclose his/her gender. Four were within the age bracket of 22-27, 44 were within the age bracket of 28-32, 18 were within the age bracket of 33-37, three were within the age bracket of 38-43 and two were within the age bracket of 44-49. Twenty respondents were in emergency medicine, 12 in family medicine, 19 in internal medicine, 10 in neurology and 10 in neurosurgery. Twenty-one were in their first year of training (PGY-1), 23 were enrolled in PGY-2, 19 were in PGY-3, seven were in PGY-4 and one was in PGY-5 (Table 1).

There were no residents in PGY-5, PGY-6 or PGY-7 in 2018 and no residents in PGY-6 or PGY-7 in 2019. Also, no residents were within the age bracket of 44-49 or 49+ in 2018 and no residents were within the age bracket of 49+ in 2019.

Burnout 2018

There was no statistical significance found amongst any subgroups in 2018. Female burnout scores tended to be higher compared with males and burnout scores tended to increase with age, however, this did not meet statistical significance. Internal medicine residents were found to have the highest burnout scores at 5.44 and neurosurgery residents had the lowest at three but not reaching statistical significance. The burnout score average for 56 medical residents in 2018 was 4.39 (Table 2).

**Table 2:** Burnout in 2018 and 2019. Burnout scores increased over time. In 2019, burnout scores for internal medicine were the highest, reaching statistical significance (t = 3.74, p = 0.001), while lowest scores for neurosurgery and reaching statistical significance in 2019 (t = -3.07, p = 0.01). PGY: Postgraduate Year

Category	Subgroup	Burnout									
		2018					2019				
		N	Mean	SD	t-test	P value	N	Mean	SD	t-test	P value
Gender											
	Male	33	4.09	2.27	-0.75	0.45	39	4.84	2.17	-0.73	0.47
	Female	21	4.66	2.52	0.5	0.62	31	5.48	2.06	1.03	0.31
	Prefer not to disclose	2	6.5	0.71	4.22	0.14	1	3			
Age											
	22-27	8	4.38	2.67	-0.01	0.988	4	6	2.31	0.78	0.49
	28-32	31	4.16	2.24	-0.48	0.62	44	5.11	2.05	0.32	0.75
	33-37	12	4.75	2.6	0.48	0.64	18	5.27	1.99	0.38	0.71
	38-43	5	4.8	2.59	0.35	0.74	3	7	1.73	1.9	0.19
	44-49	0					2	1	0		
	49+	0					0				
Specialty											
	Emergency Medicine	11	3.91	2.26	-0.71	0.49	20	4.9	1.86	-0.48	0.64
	Family Medicine	13	4.46	2.14	0.12	0.91	12	5.66	1.72	1.13	0.28
	Internal Medicine	16	5.44	2.1	1.99	0.06	19	6.31	1.42	3.74	0.001
	Neurology	9	4.11	2.52	-0.33	0.75	10	4.6	2.63	-0.6	0.56
	Neurosurgery	7	3	2.83	-1.3	0.24	10	3	2.16	-3.07	0.01
PGY											
	PGY-1	23	4.17	2.61	-0.39	0.69	21	5	2.17	-0.21	0.83
	PGY-2	25	4.8	2.04	1	0.32	23	5.34	1.72	0.69	0.49
	PGY-3	7	3.75	2.71	-0.38	0.72	19	5.1	2.38	0.01	0.99
	PGY-4	1	2	Not computed	Not computed	Not computed	7	5.14	2.54	0.04	0.96
	PGY-5	0					1	1	Not computed	Not computed	Not computed
	PGY-6	0					0				
	PGY-7	0					0				
TOTAL		56	4.39	2.35			71	5.1	2.13		

Burnout 2019

In 2019, t-test and p-values were found to be significant for internal medicine (t = 3.74, p = 0.001) and neurosurgery (t = -3.07, p = 0.01). Female burnout scores tended to be high than males but not reaching statistical significance. Neurosurgery had the lowest burnout score at 3 and internal medicine had the highest burnout score at 6.31. The burnout score average for 71 medical residents in 2019 was 5.1 which is higher compared to 2018. Across all subgroups from 2018 to 2019, burnout scores increase with the exception on neurosurgery, which remained stable (Table 2).

Optimism 2018

There was no statistical significance for optimism amongst any subgroups in 2018. PGY-1 had the lowest calculated optimism score behind the one respondent from PGY-4. The mean optimism score for all 56 residents was 16.7 with a standard deviation of 5.02 (Table 3).

**Table 3:** Optimism in 2018 and 2019. Optimism scores remained constant over time without significant differences on subgroup analysis. PGY: Postgraduate Year

Category	Subgroup	Optimism									
		2018					2019				
		N	Mean	SD	t-test	P value	N	Mean	SD	t-test	P value
Gender											
	Male	33	17.03	5.08	0.37	0.71	39	17.28	4.04	0.81	0.42
	Female	21	17.14	4.17	0.48	0.63	31	15.87	4.22	-1.17	0.25
	Prefer not to disclose	2	7.5	6.36	-2.04	0.29	1	24			
Age											
	22-27	8	16.63	6.3	-0.03	0.97	4	13.25	3.3	-2.12	0.12
	28-32	31	17.03	4.47	0.65	0.51	44	17.11	4.12	-0.05	0.96
	33-37	12	14.5	5.66	-1.34	0.21	18	17.44	4.06	0.71	0.48
	38-43	5	19.2	3.96	1.41	0.23	3	16.66	8.08	-0.02	0.99
	44-49	0					2	18.5	2.12	1.16	0.45
	49+	0					0				
Specialty											
	Emergency Medicine	11	17.55	4.11	0.68	0.51	20	18.3	3.56	1.94	0.07
	Family Medicine	13	17.31	4.68	0.47	0.65	12	16.33	4.36	-0.34	0.74
	Internal Medicine	16	16.13	5.7	-0.4	0.69	19	15.21	4.71	-1.43	0.17
	Neurology	9	13.89	5.37	-1.57	0.15	10	15.5	4.55	-0.87	0.4
	Neurosurgery	7	19.43	4.08	1.77	0.12	10	18.4	2.76	1.88	0.09
PGY											
	PGY-1	23	16.18	5.18	-0.35	0.727	21	15.76	3.43	-1.33	0.19
	PGY-2	25	16.28	5.18	-0.43	0.67	23	17	4.34	0.26	0.79
	PGY-3	7	19	3.73	2.12	0.08	19	17.21	4.87	0.4	0.69
	PGY-4	1	16	Not computed			7	17.71	4.68	0.54	0.61
	PGY-5	0					1	17	Not computed		
	PGY-6	0					0				
	PGY-7	0					0				
TOTAL		56	16.7	5.02			71	16.76	4.21		

Optimism 2019

There was no statistical significance for optimism amongst any subgroups in 2019. Emergency medicine and neurosurgery had the highest optimism scores but this did not reach statistical significance. PGY-1 had the lowest calculated optimism score. The mean optimism score for all 71 residents was 16.76 with a standard deviation of 4.21 (Table 3).

Correlation between burnout & optimism in 2018

The Pearson correlation with associated p-value was calculated between burnout and optimism scores for each subgroup in 2018. There was a statistically significant negative correlation between burnout and optimism scores for 2018 (r = -0.364, p = 0.006). Within subgroup analysis, there was statistical significance for males (r = -0.42, p = .01), within the age bracket of 28-32 (r = -0.38, p = .03), for neurology (r = -0.737, p = 0.02) and for PGY-1 (r = -0.55, p = 0.006). The correlation coefficient was not computed for the subgroup of gender who preferred not to disclose or for PGY-4 since the n was 2 and 1 for each subgroup, respectively. There was a negative correlation across all subgroups except within the age bracket of 38-43. The neurology subgroup had the strongest correlation among specialties (r = -0.737, p = 0.02) and the PGY-1 year had the strongest correlation across post-graduate training year (Table 4).

**Table 4:** Burnout vs optimism in 2018. Burnout was negatively correlated with optimism in 2018. PGY: Postgraduate Year

Category	Subgroup	2018						
			Burnout		Optimism			
		N	Mean	SD	Mean	SD	Pearson Coefficient	P value
Gender								
	Male	33	4.09	2.27	17.03	5.08	-0.421	0.015
	Female	21	4.66	2.52	17.14	4.17	-0.224	0.33
	Prefer not to disclose	2	6.5	0.71	7.5	6.36	Not computed	Not computed
Age								
	22-27	8	4.38	2.67	16.63	6.3	-0.313	0.45
	28-32	31	4.16	2.24	17.03	4.47	-0.384	0.033
	33-37	12	4.75	2.6	14.5	5.66	-0.559	0.059
	38-43	5	4.8	2.59	19.2	3.96	0.322	0.598
	44-49	0						
	49+	0						
Specialty								
	Emergency medicine	11	3.91	2.26	17.55	4.11	-0.329	0.324
	Family medicine	13	4.46	2.14	17.31	4.68	-0.198	0.517
	Internal medicine	16	5.44	2.1	16.13	5.7	-0.328	0.214
	Neurology	9	4.11	2.52	13.89	5.37	-0.737	0.023
	Neurosurgery	7	3	2.83	19.43	4.08	-0.159	0.733
PGY								
	PGY-1	23	4.17	2.61	16.18	5.18	-0.553	0.006
	PGY-2	25	4.8	2.04	16.28	5.18	-0.215	0.302
	PGY-3	7	3.75	2.71	19	3.73	-0.149	0.751
	PGY-4	1	2	Not computed	16	Not computed	Not computed	Not computed
	PGY-5	0						
	PGY-6	0						
	PGY-7	0						
TOTAL		56	4.39	2.35	16.7	5.02	-0.364	0.006

Correlation between burnout & optimism in 2019

The Pearson correlation with associated p-value was calculated between burnout and optimism scores for each subgroup in 2019. There was a statistically significant negative correlation between burnout and optimism scores for 2019 (r = -0.39, p = .001). Within subgroup analysis, statistical significance was found for females (r = -0.59, p = 0.00), within the age bracket of 28-32 (r = -0.42, p = 0.004), for emergency medicine (r = -0.6, p = 0.005), for family medicine (r = -0.577, p = 0.04) and for both PGY-2 (r = -0.627, p = 0.001) and PGY-3 (r = -0.544, p = 0.016). The correlation coefficient was not computed for the subgroup of gender who preferred not to disclose, the age bracket of 44-49 or for PGY-5, since the n was 1, 2 and 1, respectively. Both neurology and PGY-4 demonstrated a positive correlation between burnout and optimism scores while all other subgroups demonstrated a negative correlation. Emergency medicine had the strongest correlation among specialties (r = -0.6, p = 0.005) and PGY-2 had the strongest correlation across post-graduate training year (r = -0.627, p = 0.001) (Table 5).

**Table 5:** Burnout vs optimism in 2019. Burnout was negatively correlated with optimism in 2019. PGY: Postgraduate Year

Category	Subgroup	2019						
			Burnout		Optimism			
		N	Mean	SD	Mean	SD	Pearson coefficient	P value
Gender								
	Male	39	4.84	2.17	17.28	4.04	-0.181	0.27
	Female	31	5.48	2.06	15.87	4.22	-0.59	0
	Prefer not to disclose	1	3		24		Not computed	Not computed
Age								
	22-27	4	6	2.31	13.25	3.3	-0.786	0.214
	28-32	44	5.11	2.05	17.11	4.12	-0.422	0.004
	33-37	18	5.27	1.99	17.44	4.06	-0.328	0.183
	38-43	3	7	1.73	16.66	8.08	-0.143	0.909
	44-49	2	1	0	18.5	2.12	Not computed	Not computed
	49+	0						
Specialty								
	Emergency medicine	20	4.9	1.86	18.3	3.56	-0.6	0.005
	Family medicine	12	5.66	1.72	16.33	4.36	-0.577	0.049
	Internal medicine	19	6.31	1.42	15.21	4.71	-0.335	0.16
	Neurology	10	4.6	2.63	15.5	4.55	0.074	0.839
	Neurosurgery	10	3	2.16	18.4	2.76	-0.317	0.372
PGY								
	PGY-1	21	5	2.17	15.76	3.43	-0.329	0.145
	PGY-2	23	5.34	1.72	17	4.34	-0.627	0.001
	PGY-3	19	5.1	2.38	17.21	4.87	-0.544	0.016
	PGY-4	7	5.14	2.54	17.71	4.68	0.242	0.601
	PGY-5	1	1	Not computed	17	Not computed	Not computed	Not computed
	PGY-6	0						
	PGY-7	0						
TOTAL		71	5.1	2.13	16.76	4.21	-0.39	0.001

Subspecialty analysis of variance

ANOVA was calculated for burnout score and optimism scores for each specialty by pooling the scores from 2018 and 2019. Burnout was found to be statistically significant in internal medicine (F = 13.27, p = 0.00) and neurosurgery (F = 13.6, p = 0.00). Optimism was found to be statistically significant in neurology (F = 4.44, p = 0.04) and neurosurgery (F = 4.15, p = 0.04). Pooled data demonstrated internal medicine had the highest burnout scores at 5.875 while family medicine had the largest change in burnout scores from 2018 to 2019 at 1.2. All burnout scores across specialties increased from 2018 to 2019 except for neurosurgery which remained stable. Neurosurgery had the highest optimism scores on pooled analysis at 18.92 while neurology had the lowest mean at 14.70. Emergency medicine and neurology increased optimism means between 2018 and 2019 while family medicine, internal medicine and neurosurgery all decreased optimism means between 2018 and 2019 (Table 6).

**Table 6:** Burnout and optimism across specialty. Combined results from 2018 and 2019 demonstrated that burnout was highest in internal medicine (F = 13.27, p = 0.00) and lowest in neurosurgery (F = 13.6, p = 0.00). Neurology was found to have the lowest scores in optimism, although increasing over time (F = 4.44, p = 0.04) while neurosurgery had the highest optimism scores (F = 4.15, p = 0.04).

Category	Subgroup	Burnout						Optimism					
		2018	2019	Pooled				2018	2019	Pooled			
		Mean			Difference	F-ratio	P Value	Mean			Difference	F-ratio	P Value
Specialty													
	Emergency medicine	3.91	4.9	4.41	0.99	0.46	0.49	17.55	18.3	17.93	0.75	3.29	0.07
	Family medicine	4.46	5.66	5.06	1.2	0.39	0.53	17.31	16.33	16.82	-0.98	0.01	0.91
	Internal medicine	5.44	6.31	5.875	0.87	13.27	0.00	16.13	15.21	15.67	-0.92	2.94	0.09
	Neurology	4.11	4.6	4.36	0.49	0.77	0.38	13.89	15.50	14.70	1.61	4.44	0.04
	Neurosurgery	3	3	3.00	0.00	13.60	0.00	19.43	18.40	18.92	-1.03	4.15	0.04
TOTAL		4.39	5.15	4.54				16.7	16.75	16.81			

Post-graduate year analysis of variance

ANOVA was calculated for burnout score and optimism scores for each post-graduate year by pooling the scores from 2018 and 2019. There was no statistical significance found in the PGY subgroups for either burnout or optimism. All subgroup burnout scores increased between 2018 and 2019. Optimism during PGY-2 increased from 2018 to 2019 while decreasing for all other subgroups (Table 7).

**Table 7:** Burnout and optimism across post-graduate year in training. There was no statistical significance across post-graduate training year although optimism did increase as post-graduate year increased. PGY: Postgraduate Year

Category	Subgroup	Burnout						Optimism					
		2018	2019	Pooled				2018	2019	Pooled			
		Mean			Difference	F-ratio	P Value	Mean			Difference	F-ratio	P Value
Specialty													
	PGY-1	4.17	5.00	4.59	0.83	0.92	0.34	16.18	15.76	15.97	-0.42	1.30	0.26
	PGY-2	4.80	5.34	5.07	0.54	0.95	0.33	16.28	17.00	16.64	0.72	0.02	0.89
	PGY-3	3.75	5.10	4.43	1.35	0.00	0.97	19.00	17.21	18.11	-1.79	2.24	0.14
TOTAL		4.39	5.15	4.69				16.7	16.66	16.91			

## Discussion

Burnout is a significant problem during medical training, and has significant implications on resident well-being and mental health. Determining the appropriate methods to address burnout stems from research on both stressors and protective factors. The prevalence of burnout is greater among residents than physicians or medical students of similar age [9]. Combined estimates demonstrate that burnout varies among clinical specialty from 35-84% [10-15]. And generally, burnout scores are higher in surgical and residencies requiring urgent treatment of patients [5, 6]. High burnout rates are alarming as burnout has been associated with numerous outcomes including depression, medical errors and lapses in professionalism [5, 10, 16]. Specific to medical residents, burnout has a significant negative impact on developmental milestones such as the “patient care” domain requiring effective methods to address burnout among residents [10].

Several factors have been associated with burnout in residents including situational, personal and professional stressors [17]. Specific examples linked to increased burnout include emotional exhaustion, anxiety during medical school, neuroticism personality trait, increased use of electronic health record and occupational stress [5, 18-20]. However, protective factors such as personal resilience, mindfulness, self-compassion, reported levels of empathy and extraverted personalities have been associated with decreased burnout [5, 10, 13, 21, 22].

Determining appropriate methods to address burnout among residents has stemmed from research on associations to include both occupational and individual interventions. Occupational interventions focus on changes in the work environment and aim to increase job support and workplace justice [23]. In a systemic review by Busireddy et al., limiting resident work hours was the most consistent factor associated with decreased burnout [24]. On the other hand, individual interventions focus on stress management to teach people how to cope with stressful environments [25].

In this prospective cross-sectional study, we found the correlation between burnout and optimism was statistically significant and negatively correlated. This finding remained constant over time and these results were consistent across nearly all subgroup analysis. This is consistent with other studies which demonstrate the negative correlation between burnout and optimism; however, it is the first study to demonstrate this relationship among medical residents as well as the first study to demonstrate the stable relationship over time [22].

Interestingly, our analysis demonstrated that neurosurgery had the lowest burnout score which is in contrast to prior studies while internal medicine consistently had the highest burnout scores [5, 6]. Further, burnout increased over time in all subgroup analysis with the exception of neurosurgery which remained stable. The change in burnout over time may in part be attributable to the fact that the number of participants increased from 2018 to 2019, reflecting a more valid generalization, however, alternative explanations should be considered. The residency programs at our institution began in 2015 and have yet to reach full capacity across specialties. As new residents begin each year, the average age of residents increases and the average post-graduate year of training increases. However, post-graduate year of training analysis of variance did not demonstrate any difference across year and there was no trend of increased burnout scores in 2018 or 2019 as age increased. These results suggest that other variables such as institutional factors may be playing a role to affect burnout and warrant further investigation.

Although the Maslach Burnout Inventory (MBI) is generally accepted as a valid measure of burnout, our study utilized a single self-reported burnout score [26]. The single self-reported measure was used to encourage resident participation through ease of use and to quantify the variable for correlational analysis. Additionally, studies have shown the validity of a single item questionnaire to measure burnout, however, the limitation should be noted [24, 25].

Our results demonstrated that optimism scores remained relatively consistent over time. This argues against the assumption that the observed increase in burnout was related to significant changes in optimism, again suggesting that other variables are at play. Importantly, the consistent optimism scores over time demonstrate the stable nature of this trait. Drawing from research in empathy, most physicians enter medicine with established personality traits molded by parents, life experience and faith, suggesting effective interventions aim at cultivating established personality traits rather than teaching new traits or techniques [27]. Thus, future studies and interventions to decrease burnout may attempt to cultivate optimism in residents.

Our study had limitations. The main limitation was the small sample size, 56 respondents in 2018 and 71 respondents in 2019. Approximately 70% of residents responded to the survey subjecting our study to selection bias. Further, the residency programs at our institution are less than five years old and may not be generalizable to most residency programs. Statistical limitations include that the T-Test was used for sub-group analysis which increased the chance of Type 1 error and finding statistical significance. This was done since ANOVA could not be used due to the small sample size. Additionally, analysis of variance was run on pooled data from subgroups of post-graduate year and specialty rather than individual analysis by year due to the small sample size. These limitations could be overcome with larger sample size in future studies.

## Conclusions

We found optimism is negatively correlated with burnout and this remains consistent over time. Future intervention studies can address burnout through cultivation of optimism, a trait established in most residents at the start of their training.
